# Pediatric septorhinoplasty: A cohort study utilizing the pediatric health information system (PHIS) database^☆^

**DOI:** 10.1016/j.jpra.2026.07.012

**Published:** 2026-07-09

**Authors:** Molly F. MacIsaac, Ryan D. Hoffman, Joshua M. Wright, Jordan N. Halsey, S. Alex Rottgers

**Affiliations:** a Division of Plastic and Reconstructive Surgery, Johns Hopkins All Children's Hospital, St. Petersburg, FL, USA; b Department of Plastic Surgery, University of South Florida, Tampa, FL, USA

**Keywords:** Pediatric rhinoplasty, Septoplasty, Septorhinoplasty, Orofacial cleft, Cleft nasal deformity, Healthcare utilization

## Abstract

**Background:**

Pediatric rhinoplasty and septoplasty are performed for both functional and reconstructive indications, including airway obstruction, trauma, and orofacial cleft (OFC)-associated nasal deformities. Despite increasing national case volume, limited data exist on utilization patterns, procedural trends, and healthcare costs in this population.

**Methods:**

Using the Pediatric Health Information System (PHIS) database, we conducted a retrospective cohort study of patients 3-17 years old who underwent septoplasty, rhinoplasty, or septorhinoplasty between 2015 and 2022. Patients were stratified by OFC diagnosis and age group (3–14 vs. 15–17 years). Sub-analyses were performed for septoplasty-only and rhinoplasty/septorhinoplasty cohorts.

**Results:**

A total of 10,762 cases were identified; 24% had OFC. Septoplasty was nearly exclusive to non-cleft patients (98%) and increased with age, reflecting deferral until skeletal maturity. Septorhinoplasty showed a sharp volume increase after age 14, with earlier use in cleft patients as part of staged reconstruction. Graft use was significantly higher in OFC cases (21% vs. 5.5%, p < 0.0001), especially for auricular and dermal/fascial grafts. Turbinate reduction was more common in non-cleft patients. Complication and readmission rates were low across all groups (<2%). Total costs were higher in the OFC cohort but were comparable between cleft and non-cleft patients undergoing rhinoplasty/septorhinoplasty, suggesting procedure type was the primary cost driver.

**Conclusion:**

Pediatric septoplasty and rhinoplasty are safe, low-complication procedures across cleft and non-cleft populations. Trends reflect ongoing caution around timing of intervention, greater surgical complexity in cleft patients, and the need for standardized care pathways that balance functional, aesthetic, and developmental goals.

## Introduction

Pediatric rhinoplasty and septoplasty encompass a spectrum of functional and reconstructive procedures performed for nasal airway obstruction, trauma, and congenital deformities like orofacial clefts (OFC).^1^, ^2^, ^3^, ^4^ Unlike adult aesthetic rhinoplasty, pediatric nasal surgery must account for ongoing facial growth, psychosocial development, and the complexity of associated syndromic conditions.^2^^,^^5^^,^^6^ Thus, the timing and scope of intervention remain subjects of clinical debate.

In non-cleft patients, concerns regarding disruption of septal growth centers have historically prompted deferral of septoplasty until adolescence.^6^, ^7^, ^8^ In contrast, cleft nasal reconstruction is typically staged, with limited tip rhinoplasty performed in early childhood and definitive septorhinoplasty undertaken closer to skeletal maturity.^9^ Although these treatment principles are widely accepted, national data validating how consistently they are applied in contemporary practice are limited.^10^ Furthermore, pediatric patients with craniofacial diagnoses often require multidisciplinary care and may incur greater resource utilization, yet the real-world cost implications of these procedures have not been well characterized across institutions.^2^^,^^3^^,^^11^^,^^12^

Large multicenter administrative databases offer an opportunity to benchmark procedural timing, surgical complexity, and associated healthcare utilization at a national level. Using the Pediatric Health Information System (PHIS), a consortium of over 50 U.S. children’s hospitals, we sought to (1) characterize demographic and procedural patterns in pediatric septoplasty and rhinoplasty, (2) compare practice patterns between cleft and non-cleft populations, and (3) evaluate associated healthcare utilization and costs. By validating national trends in timing, technique, and associated resource utilization, this study provides benchmark data to contextualize contemporary pediatric nasal surgery practice.

## Methods

### Data source

This retrospective cohort study was approved by the Johns Hopkins Institutional Review Board (JHACH: IRB00145512) and reported in accordance with the Strengthening the Reporting of Observational Studies in Epidemiology (STROBE) guidelines. The PHIS database is a multicenter administrative database that collects de-identified clinical and resource utilization data from over 50 U.S. children’s hospitals. PHIS was queried for pediatric patients (<18 years) who underwent septoplasty, rhinoplasty, or septorhinoplasty between 2015 and 2022 using CPT procedural codes (Supplemental Table 1). Demographics, comorbidities, complications, length of stay, medication utilization (antibiotics, steroids, narcotics, tranexamic acid), and total encounter costs were extracted. Comorbidities were defined using PHIS Complex Chronic Condition (CCC) classifications. Complications were identified using PHIS-defined surgical and medical complication flags.^13^ Thirty-day readmissions were captured as defined within PHIS. Total and adjusted total encounter costs were extracted as defined within PHIS and reflect hospital-reported facility costs for the index encounter. Adjusted cost variables provided by PHIS were used to facilitate standardized comparisons across institutions. CPT codes were used to determine rhinoplasty type, graft use, and turbinate reduction. OFC status was determined using ICD-10 diagnostic codes (Supplemental Table 2). Syndromic cleft diagnoses were not analyzed separately.

Many cleft surgeons perform variable types of tip rhinoplasty at the time of primary cheiloplasty, but practice patterns and techniques widely vary. Furthermore, nasal surgery is covered under the CPT codes for cheiloplasty (CPT 40700, 40701, 40702) but some surgeons/institutions will still utilize a primary rhinoplasty code at the time of this procedure. These circumstances make the use of CPT codes for nasal surgery an unreliable indicator of what form of nasal surgery was completed in the youngest cleft population who are undergoing primary cleft lip and palate surgeries. To eliminate this uncertainty from the defined study population, patients younger than 3 years were excluded to eliminate cases representing primary cleft lip/nasal repair rather than isolated septoplasty or rhinoplasty. Patients with concurrent CPT codes for primary cleft lip/nasal repair were also excluded. Patients were stratified by presence or absence of an OFC diagnosis and by age group (3–14 and 15–17 years). Age 15 was selected as the threshold based on established literature indicating that nasal growth approaches completion during mid-to-late adolescence and that septoplasty and nasal osteotomies are more commonly performed after this stage of development.^14^ Sub-analyses were conducted for isolated septoplasty and for rhinoplasty/septorhinoplasty cohorts.

**Fig. 1:**
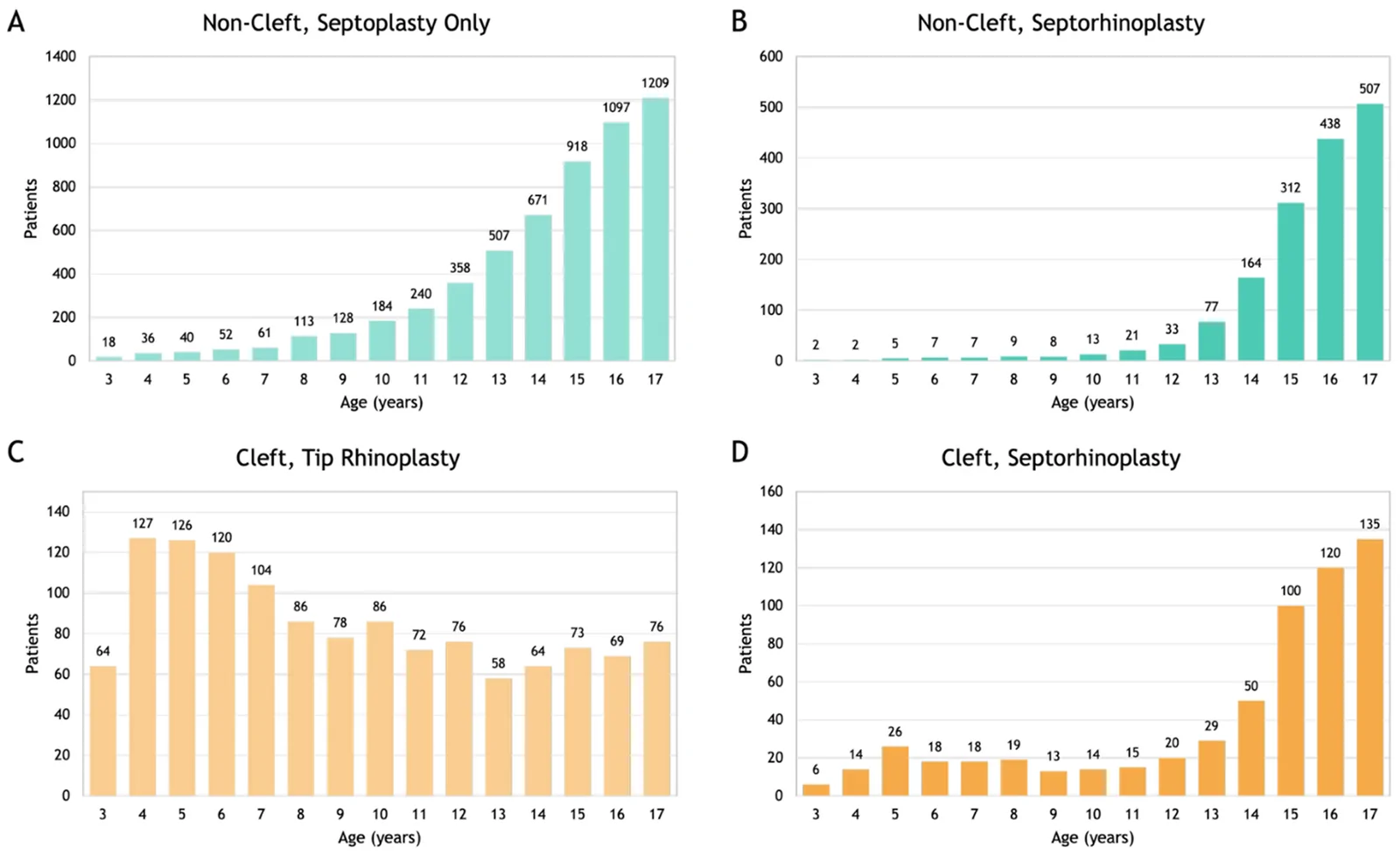
Age distribution of pediatric nasal procedures stratified by cleft status and procedure type. (A) Non-cleft patients undergoing septoplasty only (CPT 30520). (B) Non-cleft patients undergoing septorhinoplasty (CPT 30420). (C) Cleft patients undergoing tip rhinoplasty (CPT 30460). (D) Cleft patients undergoing septorhinoplasty (CPT 30462).

For visualization of age-related procedural trends, patients were stratified by cleft status and by CPT codes representing distinct procedural types (Fig. 1). In patients without a cleft diagnosis, isolated septoplasty was defined as CPT 30520 without concurrent rhinoplasty or septorhinoplasty codes (Fig. 1a), and primary septorhinoplasty was defined by CPT 30420 (Fig. 1b). In patients with a cleft diagnosis, procedures were categorized using cleft-specific rhinoplasty CPT codes, including cleft tip rhinoplasty (CPT 30460; Fig. 1c) and cleft septorhinoplasty (CPT 30462; Fig. 1d). These groupings were selected to reflect procedures with differing reconstructive scopes and typical timing across childhood and adolescence.

### Statistical analysis

Statistical analysis was performed using SAS software (Version 9.4; SAS Institute Inc, Cary, NC, USA). Two-way independent t-testing was used to analyze parametric, continuous data, and chi-square or Fisher’s exact tests were used for categorical data. Statistical significance was defined as p < 0.05. Post hoc analysis was conducted with pairwise chi-square tests for categorical data, and Bonferroni adjustment was used to determine significance.

## Results

### Demographics

A total of 10,762 pediatric rhinoplasty/septoplasty cases were identified. The mean age was 13.5 years (±3.7) and 24% had an OFC diagnosis. Most patients were male (57%), White (69%), and non-Hispanic (73%). Most procedures were covered by private (51%) or government insurance (47%). The predominant surgical setting was ambulatory surgery (84%). By cleft diagnosis, OFC patients were younger (11.1 vs. 14.2 years, p < 0.0001) and had a more balanced gender distribution more balanced gender distribution (49% female vs. 41%, p < 0.0001). White race was less common in the OFC group (64% vs. 70%, p < 0.0001), while Black (7.6% vs. 5.3%, p < 0.0001) and Asian/Pacific Islander (10% vs. 3.2%, p < 0.0001) patients were more represented (p < 0.0001). Insurance coverage did not differ between groups. Non-OFC patients had higher rates of ambulatory surgery (89% vs. 71%, p < 0.0001) while OFC patients had more observation unit stays (25% vs. 9.6%, p < 0.0001). By age group, ambulatory surgery was more frequent in older patients (87% vs. 83%, p < 0.0001), while younger patients had higher rates of observation unit stays (16% vs. 11%, p < 0.0001) (Table 1).

**Table 1:** Demographics of Pediatric Rhinoplasties by Cleft Diagnosis and Age Group.

		Comparison by Cleft Diagnosis				Comparison by Age Group			
	Total	No Cleft	Cleft	*p*	Post hoc*	3-14 years	15-17 years	*p*	Post hoc*
**Total cases**	**10762**	**8204**	**2558**			**4891**	**5871**		
**Age***mean (±SD)*	13.5 (±3.7)	14.2 (±3.0)	11.1 (±4.6)	**<.0001**		10.3 (±3.4)	16.1 (±0.8)	**<.0001**	
**Age Group,***no. (%)*				**<.0001**				**-**	
3-14 years	4891 (45)	3219 (39)	1672 (65)			-	-		
15-17 years	5871 (55)	4985 (61)	886 (35)			-	-		
**Cleft Diagnosis,***no. (%)*				**-**				**<.0001**	
No cleft	8204 (76)	-	-			3219 (66)	4985 (85)		
Cleft	2558 (24)	-	-			1672 (34)	886 (15)		
**Gender**, *no. (%)*				**<.0001**				**<.0001**	
Male	6116 (57)	4820 (59)	1296 (51)			2904 (59)	3212 (55)		
Female	4646 (43)	3384 (41)	1262 (49)			1987 (41)	2659 (45)		
**Race**, *no. (%)*				**<.0001**				**<.0001**	
White	7397 (69)	5753 (70)	1644 (64)		**<.0001**	3250 (66)	4147 (71)		**<.0001**
Other/Mixed	1578 (15)	1279 (16)	299 (12)		**<.0001**	679 (14)	899 (15)		0.037
Black	632 (5.9)	438 (5.3)	194 (7.6)		**<.0001**	360 (7.4)	272 (4.6)		**<.0001**
Asian/Pacific Islander	525 (4.9)	259 (3.2)	266 (10)		**<.0001**	300 (6.1)	225 (3.8)		**<.0001**
American Indian	29 (0.27)	24 (0.29)	5 (0.20)		0.408	10 (0.20)	19 (0.32)		0.235
Missing/Unknown	601 (5.6)	451 (5.5)	150 (5.9)		0.481	292 (6.0)	309 (5.3)		0.112
**Ethnicity**, *no. (%)*				**0.037**				0.299	
Non-Hispanic	7826 (73)	5920 (72)	1906 (75)		0.020	3591 (73)	4235 (72)		
Hispanic	2458 (23)	1903 (23)	555 (22)		0.115	1084 (22)	1374 (23)		
Missing/Unknown	478 (4.4)	381 (4.6)	97 (3.8)		0.068	216 (4.4)	262 (4.5)		
**Payment**, *no. (%)*				0.761				0.0946	
Private	5448 (51)	4166 (51)	1282 (50)			2439 (50)	3009 (51)		
Government	5043 (47)	3829 (47)	1214 (47)			2340 (48)	2703 (46)		
Other	271 (2.5)	209 (2.5)	62 (2.4)			112 (2.3)	159 (2.7)		
**Location**, *no. (%)*				**<.0001**				**<.0001**	
Ambulatory surgery	96 (0.89)	48 (0.59)	48 (1.9)		**<.0001**	56 (1.1)	40 (0.68)		**0.011**
Observation unit	9091 (84)	7266 (89)	1825 (71)		**<.0001**	3988 (82)	5103 (87)		**<.0001**
Inpatient	1442 (13)	791 (9.6)	651 (25)		**<.0001**	783 (16)	659 (11)		**<.0001**
Other	133 (1.2)	99 (1.2)	34 (1.3)		0.625	64 (1.3)	69 (1.2)		0.533

Bold denotes statistical significance.

⁎ Post hoc p-values reflect Bonferroni-adjusted pairwise comparisons. Number (No.), Standard deviation (SD).

Among 5758 isolated septoplasty cases, most cases were males (63%), and only 2.2% had an OFC diagnosis. In the age-based analysis, the majority (57%) of cases occurred in patients 15–17 years old, and ambulatory surgery was more common in older patients (91% vs. 88%, p < 0.0001), while younger patients had higher rates of observation unit stays (10% vs. 6.7%, p < 0.0001) (Table 2). Among 5004 rhinoplasty/septorhinoplasty cases, the groups were relatively evenly split in terms of OFC diagnosis (OFC: 49%), age group (younger: 48%), and gender (male: 49%). When comparing by OFC diagnosis, patients with OFC were younger (11.0 vs. 14.3 years, p < 0.0001) and more frequently Black (8% vs. 3.8%, p < 0.0001) or Asian/Pacific Islander (10% vs. 3.8%, p < 0.0001). In the age-based analysis, ambulatory surgery was more frequent in older patients (81% vs. 72%, p < 0.0001), while younger patients had higher rates of observation unit stays (22% vs. 17%, p < 0.0001) (Table 3).

**Table 2:** Septoplasty Only Sub-Analysis: Demographics by Cleft Diagnosis and Age Group.

		Comparison by Cleft Diagnosis				Comparison by Age Group			
	Total	No Cleft	Cleft	*p*	Post hoc*	3-14 years	15-17 years	*p*	Post hoc*
**Total cases**	**5758**	**5632**	**126**			**2474**	**3284**		
**Age***mean (±SD)*	14.1 (±2.9)	14.2 (±2.9)	13.1 (±3.7)	**0.001**		11.6 (±2.6)	16.1 (±0.8)	**<.0001**	
**Age Group,***no. (%)*				**0.031**				**-**	
3-14 years	2474 (43)	2408 (43)	66 (52)			-	-		
15-17 years	3284 (57)	3224 (57)	60 (48)			-	-		
**Cleft Diagnosis,***no. (%)*				-				**0.031**	
No cleft	5632 (98)	-	-			2408 (97)	3224 (98)		
Cleft	126 (2.2)	-	-			66 (2.7)	60 (1.8)		
**Gender**, *no. (%)*				0.146				**<.0001**	
Male	3646 (63)	3574 (63)	72 (57)			1640 (66)	2006 (61)		
Female	2112 (37)	2058 (37)	54 (43)			834 (34)	1278 (39)		
**Race**, *no. (%)*				**<.0001**				0.094	
White	4063 (71)	3969 (70)	94 (75)		0.314	1748 (71)	2315 (70)		
Other/Mixed	830 (14)	818 (15)	12 (9.5)		0.114	330 (13)	500 (15)		
Black	342 (5.9)	341 (6.1)	1 (0.79)		0.014	166 (6.7)	176 (5.4)		
Asian/Pacific Islander	183 (3.2)	169 (3.0)	14 (11)		**<.0001**	85 (3.4)	98 (3.0)		
American Indian	18 (0.31)	18 (0.32)	0 (0)		0.525	6 (0.24)	12 (0.37)		
Missing/Unknown	322 (5.6)	317 (5.6)	5 (4.0)		0.423	139 (5.6)	183 (5.6)		
**Ethnicity**, *no. (%)*				**0.005**				0.529	
Non-Hispanic	4267 (74)	4158 (74)	109 (87)		**0.001**	1850 (75)	2417 (74)		
Hispanic	1225 (21)	1212 (22)	13 (10)		**0.002**	509 (21)	716 (22)		
Missing/Unknown	266 (4.6)	262 (4.7)	4 (3.2)		0.435	115 (4.6)	151 (4.6)		
**Payment**, *no. (%)*				0.072				0.455	
Private	3069 (53)	2990 (53)	79 (63)			1301 (53)	1768 (54)		
Government	2553 (44)	2507 (45)	46 (37)			1109 (45)	1444 (44)		
Other	136 (2.4)	135 (2.4)	1 (0.79)			64 (2.6)	72 (2.2)		
**Location**, *no. (%)*				**<.0001**				**<.0001**	
Ambulatory surgery	5180 (90)	5091 (90)	89 (71)		**<.0001**	2177 (88)	3003 (91)		**<.0001**
Observation unit	470 (8.2)	440 (7.8)	30 (24)		**<.0001**	249 (10)	221 (6.7)		**<.0001**
Inpatient	70 (1.2)	65 (1.2)	5 (4.0)		**0.004**	35 (1.4)	35 (1.1)		0.232
Other	38 (0.66)	36 (0.64)	2 (1.6)		0.194	13 (0.53)	25 (0.76)		0.274

Bold denotes statistical significance.

⁎ Post hoc p-values reflect Bonferroni-adjusted pairwise comparisons. Number (No.), Standard deviation (SD).

**Table 3:** Rhinoplasty/Septorhinoplasty Sub-Analysis: Demographics by Cleft Diagnosis and Age Group.

		Comparison by Cleft Diagnosis				Comparison by Age Group			
	Total	No Cleft	Cleft	*p*	Post hoc*	3-14 years	15-17 years	*p*	Post hoc*
**Total cases**	**5004**	**2572**	**2432**			**2417**	**2587**		
**Age***mean (±SD)*	12.7 (±4.4)	14.3 (±3.4)	11.0 (±4.6)	**<.0001**		9.0 (±3.6)	16.1 (±0.8)	**<.0001**	
**Age Group,***no. (%)*				**<.0001**				**-**	
3-14 years	2417 (48)	811 (32)	1606 (66)			-	-		
15-17 years	2587 (52)	1761 (68)	826 (34)			-	-		
**Cleft Diagnosis,***no. (%)*				-				**<.0001**	
No cleft	2572 (51)	-	-			811 (34)	1761 (68)		
Cleft	2432 (49)	-	-			1606 (66)	826 (32)		
**Gender**, *no. (%)*				0.183				**<.0001**	
Male	2470 (49)	1246 (48)	1224 (50)	0		1264 (52)	1206 (47)		
Female	2534 (51)	1326 (52)	1208 (50)	0		1153 (48)	1381 (53)		
**Race**, *no. (%)*				**<.0001**				**<.0001**	
White	3334 (67)	1784 (69)	1550 (64)	0	**<.0001**	1502 (62)	1832 (71)		**<.0001**
Other/Mixed	748 (15)	461 (18)	287 (12)	0	**<.0001**	349 (14)	399 (15)		0.329
Black	290 (5.8)	97 (3.8)	193 (7.9)	0	**<.0001**	194 (8.0)	96 (3.7)		**<.0001**
Asian/Pacific Islander	342 (6.8)	90 (3.5)	252 (10)	0	**<.0001**	215 (8.9)	127 (4.9)		**<.0001**
American Indian	11 (0.22)	6 (0.23)	5 (0.21)	0	0.834	4 (0.17)	7 (0.27)		0.428
Missing/Unknown	279 (5.6)	134 (5.2)	145 (6.0)	0	0.246	153 (6.3)	126 (4.9)		0.025
**Ethnicity**, *no. (%)*				**<.001**				0.377	
Non-Hispanic	3559 (71)	1762 (69)	1797 (74)		**<.0001**	1741 (72)	1818 (70)		
Hispanic	1233 (25)	691 (27)	542 (22)		**<.001**	575 (24)	658 (25)		
Missing/Unknown	212 (4.2)	119 (4.6)	93 (3.8)	0	0.159	101 (4.2)	111 (4.3)		
**Payment**, *no. (%)*				**0.028**				**0.006**	
Private	2379 (48)	1176 (46)	1203 (49)	0	**0.008**	1138 (47)	1241 (48)		0.530
Government	2490 (50)	1322 (51)	1168 (48)	0	0.017	1231 (51)	1259 (49)		0.109
Other	135 (2.7)	74 (2.9)	61 (2.5)	0	0.421	48 (2.0)	87 (3.4)		**0.003**
**Location**, *no. (%)*				**<.0001**				**<.0001**	
Ambulatory surgery	3911 (78)	2175 (85)	1736 (71)		**<.0001**	1811 (75)	2100 (81)		**<.0001**
Observation unit	972 (19)	351 (14)	621 (26)		**<.0001**	534 (22)	438 (17)		**<.0001**
Inpatient	63 (1.3)	34 (1.3)	29 (1.2)		0.681	29 (1.2)	34 (1.3)		0.717
Other	58 (1.2)	12 (0.47)	46 (1.9)		**<.0001**	43 (1.8)	15 (0.6)		**<.0001**

Bold denotes statistical significance.

⁎ Post hoc p-values reflect Bonferroni-adjusted pairwise comparisons. Number (No.), Standard deviation (SD).

### Procedure details

Graft use was more common in OFC patients (21% vs. 5.5%, p < 0.0001), particularly for ear (7.0% vs. 1.1%, p < 0.0001) and rib (3.1% vs. 1.0%, p < 0.0001) grafts. Turbinate reduction was more frequent in non-OFC patients (58% vs. 23%, p < 0.0001). Comorbidities were more prevalent in the OFC group, with complex chronic conditions (55% vs. 29%, p < 0.0001) and respiratory comorbidities (23% vs. 7.6%, p < 0.0001) occurring more frequently. Surgical complications were higher in OFC cases (1.3% vs. 0.9%, p = 0.002), though overall 30-day readmission rates remained low. Mean length of stay was approximately 1 day across cohorts, with small but statistically significant differences by cleft status and age group. Perioperative medication utilization was common across cohorts, including antibiotics (86%), narcotics (98%), and steroids (87%), with minor differences by cleft status. OFC cases demonstrated higher total and adjusted encounter costs in aggregate analyses (all p < 0.05) (Table 4).

**Table 4:** Clinical Details of Pediatric Rhinoplasties by Cleft Diagnosis and Age Group.

		Comparison by Cleft Diagnosis			Comparison by Age Group		
	Total	No Cleft	Cleft	*p*	3-14 years	15-17 years	*p*
**Total cases**	**10762**	**8204**	**2558**		**4891**	**5871**	
**Graft Use, any***no. (%)*	991 (9.2)	448 (5.5)	543 (21)	**<.0001**	467 (9.5)	524 (8.9)	0.266
20912: Nasal septum	283 (2.6)	202 (2.5)	81 (3.2)	0.052	87 (1.8)	196 (3.3)	**<.0001**
21235: Auricular	272 (2.5)	92 (1.1)	180 (7.0)	**<.0001**	173 (3.5)	99 (1.7)	**<.0001**
21230/20910: Costochondral	166 (1.5)	86 (1.0)	80 (3.1)	**<.0001**	58 (1.2)	108 (1.8)	**0.006**
21210: Face bone	141 (1.3)	36 (0.44)	105 (4.1)	**<.0001**	93 (1.9)	48 (0.82)	**<.0001**
15770: Derma/fat/fascia	97 (0.90)	11 (0.13)	86 (3.4)	**<.0001**	47 (0.96)	50 (0.85)	0.550
15769: Other soft tissue	53 (0.49)	14 (0.17)	39 (1.5)	**<.0001**	21 (0.43)	32 (0.55)	0.393
15771/3/4: Liposuction	41 (0.38)	22 (0.27)	19 (0.74)	**0.001**	12 (0.25)	29 (0.49)	**0.0371**
15777: Allograft	2 (0.02)	0 (0)	2 (0.08)	0.057	1 (0.02)	1 (0.02)	1.00
**Turbinate Excision**, *no. (%)*	4967 (46)	4715 (57)	252 (10)	**<.0001**	1773 (36)	3194 (54)	**<.0001**
**CPT Rhinoplasty Code**							
30400: 1° tip rhinoplasty	310 (2.9)	241 (2.9)	69 (2.7)	0.526	199 (4.1)	111 (1.9)	**<.0001**
30410: 1° bony rhinoplasty	136 (1.3)	122 (1.5)	14 (0.55)	**<.001**	51 (1.0)	85 (1.4)	0.061
30420: 1° septorhinoplasty	1808 (17)	1605 (20)	203 (7.9)	**<.0001**	415 (8.5)	1393 (24)	**<.0001**
30430: 2° tip rhinoplasty	158 (1.5)	61 (0.74)	97 (3.8)	**<.0001**	106 (2.2)	52 (0.89)	**<.0001**
30435: 2° bony rhinoplasty	44 (0.41)	31 (0.38)	13 (0.51)	0.367	15 (0.31)	29 (0.49)	0.130
30450: 2° septorhinoplasty	118 (1.1)	61 (0.74)	57 (2.2)	**<.0001**	41 (0.84)	77 (1.3)	**0.019**
30460: Cleft tip rhinoplasty	1402 (13)	123 (1.5)	1279 (50)	**<.0001**	1163 (24)	239 (4.1)	**<.0001**
30462: Cleft septorhinoplasty	650 (6.0)	53 (0.65)	597 (23)	**<.0001**	262 (5.4)	388 (6.6)	**0.007**
30465: Vestibular stenosis	354 (3.3)	299 (3.6)	55 (2.2)	**<.001**	135 (2.8)	287 (4.9)	**0.005**
30520: Septoplasty	6425 (60)	5979 (73)	446 (17)	**<.0001**	2715 (56)	3710 (63)	**<.0001**
**Comorbidities**, *no. (%)*							
Complex chronic condition	2414 (22)	1010 (12)	1404 (55)	**<.0001**	1396 (29)	1018 (17)	**<.0001**
Respiratory	1841 (17)	518 (6.3)	1323 (52)	**<.0001**	1135 (23)	706 (12)	**<.0001**
Cardiovascular	110 (1.0)	79 (1.0)	31 (1.2)	0.274	57 (1.2)	53 (0.90)	0.177
**Complications**, *no. (%)*							
30-day readmission	137 (1.3)	117 (1.4)	20 (0.78)	**0.011**	68 (1.4)	69 (1.2)	0.322
Surgical complication	102 (0.95)	53 (0.65)	49 (1.9)	**<.0001**	62 (1.3)	40 (0.68)	**0.002**
Medical complication	3 (0.03)	3 (0.04)	0 (0)	1.00	3 (0.06)	0 (0)	0.094
**Drugs Used**, *no. (%)*							
Antibiotic	9222 (86)	6757 (82)	2465 (96)	**<.0001**	4181 (85)	5041 (86)	0.576
Narcotic	10565 (98)	8039 (98)	2526 (99)	**0.012**	4827 (99)	5738 (98)	**<.001**
Steroid	9378 (87)	7239 (88)	2139 (84)	**<.0001**	4251 (87)	5127 (87)	0.524
Tranexamic acid (TXA)	140 (1.3)	107 (1.3)	33 (1.3)	0.956	41 (0.84)	99 (1.7)	**<.001**
**LOS**, *mean (±SD)*	1.08 (±1.9)	1.09 (±2.2)	1.04 (±0.2)	**0.024**	1.13 (±2.8)	1.03 (±0.4)	**0.018**
**Costs**, *mean (±SD)*							
Total cost	7002 (±12813)	6778 (±14359)	7719 (±5363)	**<.0001**	6954 (±17971)	7041 (±5650)	0.745
Adjusted total	6866 (±12107)	6609 (±13541)	7691 (±5269)	**<.0001**	6882 (±17010)	6853 (±5261)	0.907

Bold denotes statistical significance.

Current Procedural Terminology (CPT), Length of stay (LOS), Number (No.), Standard deviation (SD).

Among 5758 isolated septoplasty cases, the majority of cases occurred in the non-OFC population (98%). Graft use was uncommon (0.7%) and turbinate reduction was frequent (65%). Surgical complications (0.6%), 30-day readmissions (1.6%), and medical complications (0.05%) were rare (Table 5). Among 5004 rhinoplasty/septorhinoplasty cases, graft use was common (19%) and more frequent in patients with OFC (22% vs. 16%, p < 0.0001). Nasal septal grafts were more common in non-OFC patients (7.4% vs 3.2%, p < 0.0001) while auricular cartilage grafts were more common in OFC cases (7.4% vs. 3.3%). Costochondral grafts were used similarly between cohorts (3.2% each). Turbinate reduction was more common in non-OFC patients (39% vs. 8.7%, p < 0.0001) and increased with age. Overall complication rates were low, though cleft patients had a higher rate of surgical complications compared to non-cleft patients (1.9% vs. 0.74%, p < 0.001) (Table 6).

**Table 5:** Septoplasty Only Sub-Analysis: Clinical Details by Cleft Diagnosis and Age Group.

		Comparison by Cleft Diagnosis			Comparison by Age Group		
	Total	No Cleft	Cleft	*p*	3-14 years	15-17 years	*p*
**Total cases**	**5758**	**5632**	**126**		**2474**	**3284**	
**Graft Use, any***no. (%)*	40 (0.69)	28 (0.50)	12 (9.5)	**<.0001**	24 (0.97)	16 (0.49)	**0.029**
20912: Nasal septum	16 (0.28)	12 (0.21)	4 (3.2)	**<.001**	9 (0.36)	7 (0.21)	0.282
21235: Auricular	7 (0.12)	6 (0.11)	1 (0.79)	0.144	4 (0.16)	3 (0.09)	0.472
21230/20910: Costochondral	6 (0.10)	4 (0.07)	2 (1.6)	**0.007**	3 (0.12)	3 (0.09)	1.00
21210: Facial bone	7 (0.12)	2 (0.04)	5 (4.0)	**<.0001**	6 (0.24)	1 (0.03)	**0.047**
15770: Derma/fat/fascia	1 (0.02)	1 (0.02)	0 (0)	1.00	1 (0.04)	0 (0)	0.430
15769: Other soft tissue	1 (0.02)	1 (0.02)	0 (0)	1.00	0 (0)	1 (0.03)	1.00
15771/3/4: Liposuction	4 (0.07)	3 (0.05)	1 (0.79)	0.085	2 (0.08)	2 (0.06)	1.00
15777: Allograft	1 (0.02)	0 (0)	1 (0.79)	**0.022**	1 (0.04)	0 (0)	0.430
**Turbinate Excision**, *no. (%)*	3742 (65)	3702 (66)	40 (32)	**<.0001**	1510 (61)	2232 (68)	**<.0001**
**Comorbidities**, *no. (%)*							
Complex chronic condition	510 (8.9)	475 (8.4)	35 (28)	**<.0001**	256 (10)	254 (7.7)	**0.001**
Respiratory	170 (3.0)	145 (2.6)	25 (20)	**<.0001**	100 (4.0)	70 (2.1)	**<.0001**
Cardiovascular	55 (0.96)	51 (0.91)	4 (3.2)	**0.032**	27 (1.1)	28 (0.85)	0.357
**Complications**, *no. (%)*							
30-day readmission	90 (1.6)	89 (1.6)	1 (0.79)	0.724	45 (1.8)	45 (1.4)	0.174
Surgical complication	36 (0.63)	34 (0.60)	2 (1.6)	0.186	16 (0.65)	20 (0.61)	0.857
Medical complication	3 (0.05)	3 (0.05)	0 (0)	1.00	3 (0.12)	0 (0)	0.079
**Drugs Used**, *no. (%)*							
Antibiotic	4472 (78)	4358 (77)	114 (90)	**0.001**	1870 (76)	2602 (79)	**0.001**
Narcotic	5668 (98)	5543 (98)	125 (99)	0.724	2442 (99)	3226 (98)	0.152
Steroid	5105 (89)	4995 (89)	110 (87)	0.627	2229 (90)	2876 (88)	**0.003**
Tranexamic acid (TXA)	38 (0.66)	31 (0.55)	7 (5.6)	**<.0001**	12 (0.49)	26 (0.79)	0.155
**LOS**, *mean (±SD)*	1.12 (±2.6)	1.12 (±2.6)	1.08 (±0.3)	0.327	1.23 (±3.9)	1.04 (±0.5)	**0.020**
**Costs**, *mean (±SD)*							
Total cost	6321 (±16829)	6269 (±16972)	8631 (±7910)	**0.002**	6827 (±24805)	5940 (±5731)	0.081
Adjusted total	6194 (±15872)	6141 (±16001)	8548 (±7955)	**0.001**	6745 (±23430)	5779 (±5276)	**0.044**

Bold denotes statistical significance.

Length of stay (LOS), Number (No.), Standard deviation (SD).

**Table 6:** Rhinoplasty/Septorhinoplasty Sub-Analysis: Clinical Details by Cleft Diagnosis and Age Group.

		Comparison by Cleft Diagnosis			Comparison by Age Group		
	Total	No Cleft	Cleft	*p*	3-14 years	15-17 years	*p*
**Total cases**	**5004**	**2572**	**2432**		**2417**	**2587**	
**Graft Use, any***no. (%)*	951 (19)	420 (16)	531 (22)	**<.0001**	443 (18)	508 (20)	0.239
20912: Nasal septum	267 (5.3)	190 (7.4)	77 (3.2)	**<.0001**	78 (3.2)	189 (7.3)	**<.0001**
21235: Auricular	265 (5.3)	86 (3.3)	179 (7.4)	**<.0001**	169 (7.0)	96 (3.7)	**<.0001**
21230/20910: Costochondral	160 (3.2)	82 (3.2)	78 (3.2)	0.970	55 (2.3)	105 (4.1)	**<.001**
21210: Facial bone	134 (2.7)	34 (1.3)	100 (4.1)	**<.0001**	87 (3.6)	47 (1.8)	**<.0001**
15770: Derma/fat/fascia	96 (1.9)	10 (0.39)	86 (3.5)	**<.0001**	46 (1.9)	50 (1.9)	0.939
15769: Other soft tissue	52 (1.0)	13 (0.51)	39 (1.6)	**<.001**	21 (0.87)	31 (1.2)	0.251
15771/3/4: Liposuction	37 (0.74)	19 (0.74)	18 (0.74)	0.995	10 (0.41)	27 (1.0)	**0.009**
15777: Allograft	1 (0.02)	0 (0)	1 (0.04)	0.486	0 (0.00)	1 (0.04)	1.00
**Turbinate Excision**, *no. (%)*	1225 (24)	1013 (39)	212 (8.7)	**<.0001**	263 (11)	962 (37)	**<.0001**
**CPT Rhinoplasty Code**							
30400: 1° tip rhinoplasty	310 (6.2)	241 (9.4)	69 (2.8)	**<.0001**	199 (8.2)	111 (4.3)	**<.0001**
30410: 1° bony rhinoplasty	136 (2.7)	122 (4.7)	14 (0.58)	**<.0001**	51 (2.1)	85 (3.3)	**0.011**
30420: 1° septorhinoplasty	1808 (36)	1605 (62)	203 (8.3)	**<.0001**	415 (17)	1393 (54)	**<.0001**
30430: 2° tip rhinoplasty	158 (3.2)	61 (2.4)	97 (4.0)	**0.001**	106 (4.4)	52 (2.0)	**<.0001**
30435: 2° bony rhinoplasty	44 (0.9)	31 (1.2)	13 (0.53)	**0.011**	15 (0.62)	29 (1.1)	0.058
30450: 2° septorhinoplasty	118 (2.4)	61 (2.4)	57 (2.3)	0.948	41 (1.70)	77 (3.0)	**0.003**
30460: Cleft tip rhinoplasty	1402 (28)	123 (4.8)	1279 (53)	**<.0001**	1163 (48)	239 (9.2)	**<.0001**
30462: Cleft septorhinoplasty	650 (13)	53 (2.1)	597 (25)	**<.0001**	262 (11)	388 (15)	**<.0001**
30465: Vestibular stenosis	354 (7.1)	299 (12)	55 (2.3)	**<.0001**	135 (5.6)	219 (8.5)	**<.0001**
30520: Septoplasty	667 (13)	347 (13)	320 (13)	0.729	241 (10)	426 (16)	**<.0001**
**Comorbidities**, *no. (%)*							
Complex chronic condition	1904 (38)	535 (21)	1369 (56)	**<.0001**	1140 (47)	764 (30)	**<.0001**
Respiratory	1671 (33)	373 (15)	1298 (53)	**<.0001**	1035 (43)	636 (25)	**<.0001**
Cardiovascular	55 (1.1)	28 (1.1)	27 (1.1)	0.942	30 (1.2)	25 (0.97)	0.351
**Complications**, *no. (%)*							
30-day readmission	47 (0.94)	28 (1.1)	19 (0.78)	0.260	23 (1.0)	24 (0.9)	0.930
Surgical complication	66 (1.3)	19 (0.74)	47 (1.9)	**<.001**	46 (1.9)	20 (0.77)	**0.001**
Medical complication	0 (0)	0 (0)	0 (0)	-	0 (0)	0 (0)	-
**Drugs Used**, *no. (%)*							
Antibiotic	4750 (95)	2399 (93)	2351 (97)	**<.0001**	2311 (96)	2439 (94)	**0.032**
Narcotic	4897 (98)	2496 (97)	2401 (99)	**<.0001**	2385 (99)	2512 (97)	**<.001**
Steroid	4273 (85)	2244 (87)	2029 (83)	**<.001**	2022 (84)	2251 (87)	**0.001**
Tranexamic acid (TXA)	102 (2.0)	76 (3.0)	26 (1.1)	**<.0001**	29 (1.20)	73 (2.8)	**<.0001**
**LOS**, *mean (±SD)*	1.03 (±0.2)	1.02 (±0.2)	1.03 (±0.2)	**0.012**	1.03 (±0.2)	1.02 (±0.2)	0.231
**Costs**, *mean (±SD)*							
Total cost	7785 (±5103)	7893 (±5014)	7672 (±5195)	0.126	7085 (±4879)	8440 (±5221)	**<.0001**
Adjusted total	7640 (±4926)	7634 (±4766)	7646 (±5090)	0.931	7023 (±4864)	8216 (±4914)	**<.0001**

Bold denotes statistical significance.

Current Procedural Terminology (CPT), Length of stay (LOS), Number (No.), Standard deviation (SD).

Fig. 1 displays age distribution by procedure type and cleft status. Non-cleft septoplasty (Fig. 1a) and septorhinoplasty (Fig. 1b) volumes increased steadily with age. Cleft-related tip rhinoplasty was most common between ages 3–7 (Fig. 1c) and cleft septorhinoplasty volumes increased after age 13 (Fig. 1d).

## Discussion

Septoplasty and rhinoplasty are common procedures performed in the pediatric patient population. This study demonstrates that septoplasty and rhinoplasty are safe operations with low rates of 30-day hospital readmission (≤1.4%), surgical complications (≤1.9%), and medical complications (≤0.07%). Across all patients and all age groups, these procedures are most often performed in the outpatient setting (84%) in patients with low rates of chronic (22%), cardiovascular (1%), and respiratory (17%) comorbidities. These procedures are also more common in males (57%), possibly due to the higher incidence of OFC and trauma in this population.^15^^,^^16^ Collectively, these findings support the safety and resource profile of contemporary pediatric nasal surgery across diverse institutional settings.

These data allow for comparison of patients with a diagnosis of OFC to those without. The modern treatment paradigm of patients with OFCs involves tip rhinoplasty at an early age and/or at the time of primary lip repair, followed by definitive septorhinoplasty to correct OFC-associated deformities and nasal obstruction near the age of skeletal maturity, typically 15–18 in males and 12–16 in females.^17^^,^^18^ Compared to patients without OFCs, both septoplasty and rhinoplasty patients with OFCs have higher rates of chronic medical comorbidities (28% vs 8.4%, p < 0.001) and respiratory comorbidities (20% vs 2.6%, p < 0.0001), which is consistent with the association of OFC with many syndromic diagnoses.^19^^,^^20^

### Clinical and demographic trends

Males comprised 57% of the cohort, with variation by procedure type and cleft status. Septoplasty-only patients were predominantly male (63%). While females may experience higher rates of chronic nasal congestion due to hormonally mediated mucosal inflammation,^21^ this does not appear to drive surgical utilization. Instead, the male predominance may be related to higher rates of nasal trauma, which disproportionately affects boys and remains a common indication for pediatric septoplasty.^15^^,^^16^ In the rhinoplasty cohort, sex distribution was balanced overall (50/50), though cleft-related cases showed a nearly even male-to-female split (51% vs. 49%) despite the known 2:1 male predominance in CL/P birth incidence.^22^ This may reflect delayed surgery in males due to later skeletal maturity or lower concern about nasal appearance.

Racial representation in our cohort diverged from national birth demographics, with white patients comprising 69% of the study population compared to 51% of U.S. birth rates.^23^ Black and Asian/Pacific Islander patients were underrepresented at 5.9% and 4.9%, respectively (vs. 14% and 6.5%).^23^ These disparities varied by procedure type. In the rhinoplasty cohort, racial differences were more pronounced and closely tied to cleft status. White patients more frequently underwent non-cleft rhinoplasty, while Black and Asian/Pacific Islander patients were disproportionately represented in the cleft rhinoplasty subgroup. Several contributing factors may be postulated. First, nasal anatomy varies significantly across populations; Black and Asian patients often have features (e.g., flatter/lower dorsum, wider base, shorter columella, less projected tip, thicker skin) that may not present the same functional or cosmetic indications for non-cleft rhinoplasty as seen in white patients.^24^, ^25^, ^26^, ^27^, ^28^ Second, cleft epidemiology aligns with observed patterns: Black patients, who are about half as likely as white patients to be born with CL/P, made up 7.9% of cleft rhinoplasty cases, which is about half their national birth rate (14%).^22^ In contrast, Asian/Pacific Islander patients, who have higher CL/P rates, represented 10% of cleft rhinoplasty cases (compared to their national birth rate of 6.5%).^22^ Third, disparities in access to subspecialty care may limit timely referral and surgical intervention for some minority groups.^29^

In the septoplasty-only group, which was predominantly non-cleft, racial distribution was similar between cleft and non-cleft subgroups. However, white patients were markedly overrepresented (71% of septoplasties), and Black and Asian patients were underrepresented. Hypothetical causes of these observations include anatomical variation, where septal deviation requiring surgical correction may be more common in populations with narrower nasal structures,^24^^,^^25^^,^^27^ and/or differences in healthcare access, including timely referral to surgical specialists.^29^ Together, these factors create a complex, nonlinear relationship between race and procedure type, shaped by anatomy, epidemiology, and structural barriers to care.

### Timing of intervention and skeletal maturity

Isolated septoplasty was performed almost exclusively in non-cleft patients (98%) and increased steadily with age, with most cases occurring in late adolescence (Fig. 1a). This pattern likely reflects continued adherence to the longstanding paradigm of delaying septoplasty until nasal/midfacial growth is complete due to concerns that disruption of the nasal septum, particularly the sphenodorsal and sphenospinal growth zones, could impair midfacial development.^6^, ^7^, ^8^ However, recent literature suggests that when performed with growth-sparing techniques, septoplasty does not adversely affect nasal or facial development.^5^^,^^30^^,^^31^ Nevertheless, our data indicate that most septoplasty procedures are still performed after the age of 15, suggesting that many providers continue to defer intervention until near skeletal maturity or that this pathology more likely manifests in this older population.

In contrast, septorhinoplasty involves broader structural manipulation, including work on the nasal dorsum and tip, and carries a higher theoretical risk of growth disturbance when performed before facial maturity. Reflecting this, we found that very few septorhinoplasties were performed at younger ages, with a sharp increase in volume beginning around age 14–15 (Fig. 1b, 1d). This trend was consistent in both cleft and non-cleft groups, though cleft patients accounted for a greater proportion of early adolescent septorhinoplasty cases, possibly due to staged reconstructive planning or airway considerations. Turbinate reduction was more common in older, non-cleft patients undergoing septorhinoplasty, reflecting nasal airway obstruction as a key surgical indication in this group.

Cleft-related tip rhinoplasty was most common between ages 4–7 (Fig. 1c). These cases likely represent early intermediate nasal revisions and are consistent with the widely accepted two-stage cleft nasal reconstruction strategy. This approach balances aesthetic and psychosocial benefits in early childhood with long-term structural correction in adolescence.

### Graft utilization patterns

Grafts were more frequently used in cleft-associated septorhinoplasty compared to non-cleft cases (22% vs 16%). However, this likely underestimates the use of septal cartilage, as it is often harvested and incorporated within the septorhinoplasty procedure itself (CPT 30420, 30450, 30462) without requiring a separate CPT code. To identify remote donor sites, we therefore analyzed codes representing cartilage harvest from sites such as the rib and ear. Ear cartilage was the most frequently used in the cleft population (7.4%), occurring more than twice as often as in the non-cleft group (3.3%, P < 0.0001). Rib cartilage, while less commonly used overall (3.2% in cleft cases), offers a large volume of material with favorable structural properties, though drawbacks include postoperative pain, proximity to the pleura, and tendency to warp. Ear cartilage, despite its irregular shape and intrinsic curvature, offers the advantages of lower donor site morbidity and proximity to the operative field.^32^, ^33^, ^34^

A small but significant proportion of cleft-associated procedures (3.5% vs. 0.039%, P < 0.0001) utilized a universal graft harvest code that may represent dermal fat or fascia harvested from various donor sites. These grafts may be used for dorsal augmentation^35^, ^36^, ^37^ alar base soft tissue support,^33^^,^^34^^,^^38^ or lip revision.^39^^,^^40^ The low frequency of allograft use (<0.05%) is consistent with preference for autologous materials in this population and the availability of native septal cartilage during primary procedures.

### Healthcare costs and resource allocation

Across the national cohort, pediatric septoplasty and rhinoplasty were predominantly ambulatory procedures, with mean lengths of stay approximating one day and low 30-day readmission and complication rates. This suggests that contemporary pediatric nasal surgery is delivered within a resource-contained framework, even among medically complex cleft patients. Differences in utilization metrics were small in magnitude and largely reflected procedural complexity rather than prolonged hospitalization or excess inpatient burden.

In the overall cohort, total hospital costs were higher for patients with cleft diagnoses (Table 4). However, this appears to be driven by the higher proportion of cleft patients undergoing rhinoplasty/septorhinoplasty procedures, which are more expensive than septoplasty alone. Rhinoplasty/septorhinoplasty sub-analysis revealed that total costs were similar between cleft and non-cleft patients (Table 6), suggesting that procedure type, rather than cleft status itself, is the primary cost driver. Given that nearly all septoplasty-only cases occurred in non-cleft patients (98%), and septoplasty has lower associated costs, the overall difference in cost likely reflects differences in procedure distribution.

Several billing discrepancies were noted in a small subset of cases, likely reflecting institutional variation, inconsistent CPT interpretation, or documentation issues. One common example was co-billing of primary cleft lip repair (e.g., CPT 40700–40702) with cleft tip rhinoplasty (CPT 30460) during infancy. As current standards of care typically include nasal tip correction within the primary cleft lip procedure, this may represent inadvertent redundancy or overbilling. Similarly, we identified cases where both septoplasty (CPT 30520) and cleft septorhinoplasty (CPT 30462) were billed concurrently, despite 30462 encompassing septal work. These patterns suggest potential confusion regarding bundling rules and may warrant further clarification from CMS and specialty societies, as well as highlight the need for improved consistency in documentation and billing practices.

### Limitations

This study has several limitations inherent to retrospective database research. The accuracy of our findings depends on the fidelity of billing codes, which may be subject to miscoding or variation in provider practices. While we attempted to correct for common coding errors and unbundling, nuances in operative technique and surgeon intent cannot be fully captured through CPT and ICD codes alone. Furthermore, PHIS does not capture long-term follow-up, aesthetic outcomes, patient-reported outcomes, or longitudinal growth data; future prospective studies will be necessary to evaluate the efficacy and durability of pediatric nasal surgery across both cleft and non-cleft populations. With such a large sample size, there is the potential for overestimate effect size and demonstrate statistical significance that does not reflect clinical significance. We have attempted to highlight the differences of greatest importance in our discussion. Finally, socioeconomic variables such as income, education level, and regional access to care are not included in PHIS, which limits analysis of underlying disparities. Observed associations may be influenced by confounding variables both captured and uncaptured within the database, and further multivariable analyses may help clarify the independent contributions of these factors. Nevertheless, the large, nationally representative sample size and standardized data structure provide a valuable lens into pediatric rhinoplasty trends and highlight several targets for quality improvement, coding education, and resource allocation.

## Conclusion

Pediatric septoplasty and rhinoplasty are safe procedures with low complication and readmission rates in both cleft and non-cleft populations. Procedural patterns vary by sex and race, with trauma likely contributing to male predominance and cleft status influencing racial representation. Most septoplasties and septorhinoplasties are performed after age 15, reflecting ongoing caution around midfacial growth despite literature supporting earlier, growth-sparing approaches in septoplasties. Grafts are used more frequently and diversely in cleft cases, highlighting greater anatomical complexity. While total costs are higher in cleft patients, this difference is driven by procedure type rather than cleft status itself. These findings provide national benchmarks for procedural timing, graft utilization, and associated resource use in pediatric nasal reconstruction.

## Declarations

None declared.

## Funding

None.

## Disclosures

No financial disclosures or conflicts of interest to declare.

## Ethical approval

This study received ethical approval from the Johns Hopkins Institutional Review Board (JHACH: IRB00145512) on July 26, 2017. This is an IRB-approved retrospective study. All patient information was de-identified and patient consent was not required. Patient data will not be shared with third parties.

## Appendix Supplementary materials

Image, application 1

Image, application 2
