# Supplementary material for: Pediatric septorhinoplasty: A cohort study utilizing the pediatric health information system (PHIS) database
Source: JPRAS Open. 2026 Jul 9;51:755–69. doi: 10.1016/j.jpra.2026.07.012 (PMC13505433; doi:10.1016/j.jpra.2026.07.012)
Supplement: Supplementary file 1 [file mmc1.docx]

| **Supplemental Table 1.** CPT Code Distribution for Pediatric Rhinoplasty Procedures | | | | |
| --- | --- | --- | --- | --- |
| **CPT Code** | **Full Description** | **Short Description** | **Count** | **Percent** |
| **30400** | Primary rhinoplasty for the lateral and alar cartilages and/or tip | 1° tip rhinoplasty | 480 | 4.5% |
| **30410** | Primary rhinoplasty for the bony pyramid, lateral and alar cartilages, and/or tip | 1° bony rhinoplasty | 162 | 1.5% |
| **30420** | Primary rhinoplasty that includes major septal repair | 1° septorhinoplasty | 1873 | 17% |
| **30430** | Secondary rhinoplasty for a minor revision, such as a small amount of nasal tip work | 2° tip rhinoplasty | 172 | 1.6% |
| **30435** | Secondary rhinoplasty for an intermediate revision, such as bony work with osteotomies | 2° bony rhinoplasty | 45 | 0.4% |
| **30450** | Secondary rhinoplasty for a major revision, such as nasal tip work and osteotomies | 2° septorhinoplasty | 120 | 1.1% |
| **30460** | Rhinoplasty for a nasal deformity secondary to a congenital cleft lip and/or palate, including columellar lengthening, tip only | Cleft tip rhinoplasty | 3585 | 33% |
| **30462** | Rhinoplasty for a nasal deformity secondary to a congenital cleft lip and/or palate, including columellar lengthening, tip, septum, osteotomies | Cleft septorhinoplasty | 944 | 8.8% |
| **30465** | Repair of nasal vestibular stenosis, such as spreader grafting or lateral nasal wall reconstruction | Vestibular Stenosis | 394 | 3.7% |
| **30520** | Septoplasty or submucous resection, with or without cartilage scoring, contouring, or replacement with a graft | Septoplasty | 6677 | 62% |
| Current Procedural Terminology (CPT) | | | | |
