# Supplementary material for: Pediatric septorhinoplasty: A cohort study utilizing the pediatric health information system (PHIS) database
Source: JPRAS Open. 2026 Jul 9;51:755–69. doi: 10.1016/j.jpra.2026.07.012 (PMC13505433; doi:10.1016/j.jpra.2026.07.012)
Supplement: Supplementary file 2 [file mmc2.docx]

| **Supplemental Table 2.** ICD-CM-10 Codes for Cleft Lip and/or Palate | |
| --- | --- |
| **ICD CM 10 Code:** | **Description** |
| **Q35.1** | Cleft hard palate |
| **Q35.3** | Cleft soft palate |
| **Q35.5** | Cleft hard palate with cleft soft palate |
| **Q35.7** | Cleft uvula |
| **Q35.9** | Cleft palate, unspecified |
| **Q36** | Cleft lip, bilateral |
| **Q36.1** | Cleft lip, median |
| **Q36.9** | Cleft lip, unilateral |
| **Q37** | Cleft hard palate with bilateral cleft lip |
| **Q37.1** | Cleft hard palate with unilateral cleft lip |
| **Q37.2** | Cleft soft palate with bilateral cleft lip |
| **Q37.3** | Cleft soft palate with unilateral cleft lip |
| **Q37.4** | Cleft hard and soft palate with bilateral cleft lip |
| **Q37.5** | Cleft hard and soft palate with unilateral cleft lip |
| **Q37.8** | Unspecified cleft palate with bilateral cleft lip |
| **Q37.9** | Unspecified cleft palate with unilateral cleft lip |
| **Z87.730** | Personal history of (corrected) cleft lip and palate |
| The International Classification of Diseases, 10th Revision, Clinical Modification (ICD–10–CM) | |
